# Human adrenocortical organoids for tissue regeneration and disease modeling

**DOI:** 10.1016/j.stemcr.2025.102679

**Published:** 2025-10-16

**Authors:** Qing Li, Xiaoyu Li, Yiming Zhang, Yanting Shen, Zhiqiang Lu, Wei Chen, Yujun Liu, Shuang Wu, Xiaofeng Gong, Xuewen Li, Nicole Bechmann, Jingjing Jiang, Bing Zhao

**Affiliations:** 1 School of Basic Medical Sciences, Institute of Biomedical Innovation, The First Affiliated Hospital, Jiangxi Medical College, Nanchang University, Nanchang 330031, China; 2 Department of Endocrinology and Metabolism, Zhongshan Hospital, Fudan University, Shanghai 200032, China; 3 Department of Urology, Zhongshan Hospital, Fudan University, Shanghai 200032, China; 4 Z Lab, BioGenous BIOTECH, Shanghai 200438, China; 5 Institute of Clinical Chemistry and Laboratory Medicine, University Hospital Carl Gustav Carus, Medical Faculty Carl Gustav Carus, Technische Universität Dresden, Fetscherstrasse 74, 01307 Dresden, Germany

**Keywords:** human adrenocortical organoids, adrenal regeneration, adrenal insufficiency, adrenocortical adenoma, PRKACA L206R

## Abstract

The adrenal glands are essential endocrine organs that secrete key hormones maintaining physiological homeostasis. Herein, we established expandable three-dimensional (3D) human adrenocortical organoid (ACO) cultures that preserved the characteristic of zona fasciculata cell lineages and retained their capacity to produce cortisol. The ACOs could secrete glucocorticoids in response to physiological stimuli and thus rescue adrenalectomized mice, indicating their potential for the treatment of primary adrenal insufficiency. Furthermore, by introducing a hotspot pathogenic variant (PRKACA L206R) identified in Cushing’s syndrome, we achieved the organoid disease modeling of cortisol-producing adenomas. In summary, this study establishes a human organoid platform to explore homeostasis and dysfunction in adrenal glands, suggesting future applications in disease modeling and regenerative medicine.

## Highlights

• Adrenocortical organoids (ACOs) can be generated from normal human adrenal glands
• Human ACOs are responsive to adrenocorticotropic hormone (ACTH) and angiotensin II
• Transplantation of human ACOs rescues adrenal insufficiency in adrenalectomized mice
• Expression of PRKACA-L206R in ACOs mimics cortisol-producing adenomas

## Teaser

In this article, Zhao and colleagues established a culture system to generate expandable adrenocortical organoids (ACOs) from normal human adrenal glands. These ACOs are a useful tool for exploring adrenal physiology and can also be applied in disease modeling and tissue regeneration.

## Introduction

The adrenal gland is a vital endocrine organ comprising the outer adrenal cortex and the inner adrenal medulla beneath a common capsule (Lyraki and Schedl, 2021). The adrenal medulla produces catecholamines that mediate the acute stress response (fight-or-flight). Derived from the mesoderm, the adrenal cortex synthesizes steroid hormones and is subdivided into three distinct zones. The outermost zona glomerulosa (zG) mainly secretes mineralocorticoids, such as aldosterone, which are involved in blood pressure control. The middle zona fasciculata (zF) produces glucocorticoids (cortisol in humans and corticosterone in rodents) under the control of adrenocorticotropic hormone (ACTH). The innermost zona reticularis (zR) is capable of producing and secreting androgens. Underproduction or overproduction of steroid hormones can seriously affect the physiological function and metabolism.

Adrenal insufficiency is a disorder resulting in the absolute or relative deficiency of glucocorticoid production (Husebye et al., 2021). Currently, the only approved treatment for affected patients is glucocorticoid supplementation, which can, however, cause undesirable side effects such as high blood pressure, obesity, and diabetes mellitus if overdosed (Ueland and Husebye, 2018). Hence, for patients with adrenal insufficiency, a long-term and stable cell-replacement strategy has become an urgent clinical issue to be tackled. Several studies have utilized cell-replacement strategies to address adrenal insufficiency in adrenalectomized rodent models (Tanaka et al., 2020; Thomas et al., 2000). Nevertheless, most grafts failed to fully restore normal adrenal hormone production. Recent experiments using primary bovine cells or porcine spheroids succeeded in treating adrenal insufficiency in rodents, but these advances are unlikely to proceed to the clinical stage owing to safety concerns and because those culture conditions were not serum-free (Balyura et al., 2015; Maria et al., 2023). Consequently, there is an unmet need for suitable culture system to expand human-derived adrenocortical cells *in vitro*, which can be utilized to treat patients.

The complex regulatory network underlying human adrenal development and disease remains poorly understood due to the limitations of current models. Species-specific differences have further hindered our understanding (Kempná and Flück, 2007). For instance, the presence of *CYP17A1* in human adrenal cortex enables the production of cortisol as the major form of glucocorticoid in humans. In contrast, rodent adrenals lack *Cyp17a1* and mainly produce corticosterone instead (Weerden et al., 1992). Human organoids are stem cell-derived three-dimensional structures that mimic features of the pertinent tissue (Fujii and Sato, 2020) and have great value in disease modeling, drug screening, and regenerative medicine. To investigate human and mouse adrenal development and steroidogenesis, fetal zone adrenocortical-like cells or steroid-producing cells derived from induced pluripotent stem cells (iPSCs) have been produced (Gerard et al., 2023; Oikonomakos et al., 2024; Sakata et al., 2022; Steenblock et al., 2023). So far, adult human adrenocortical organoids (ACOs) have not yet been reported.

In this study, we established an expandable human ACOs culture system using adult human primary adrenal tissue. The ACOs mainly preserved the characteristics of zF and were capable of producing glucocorticoids in response to ACTH. Transplantation of ACOs rescued cortisol deficiency-related phenotypes in adrenalectomized mice. The ACOs also demonstrated potential as a tool for studying pathogenic variants (PVs) and related signaling pathways in adrenal tumors.

## Results

### Establishment of human adrenocortical organoid culture

**Figure 1:**
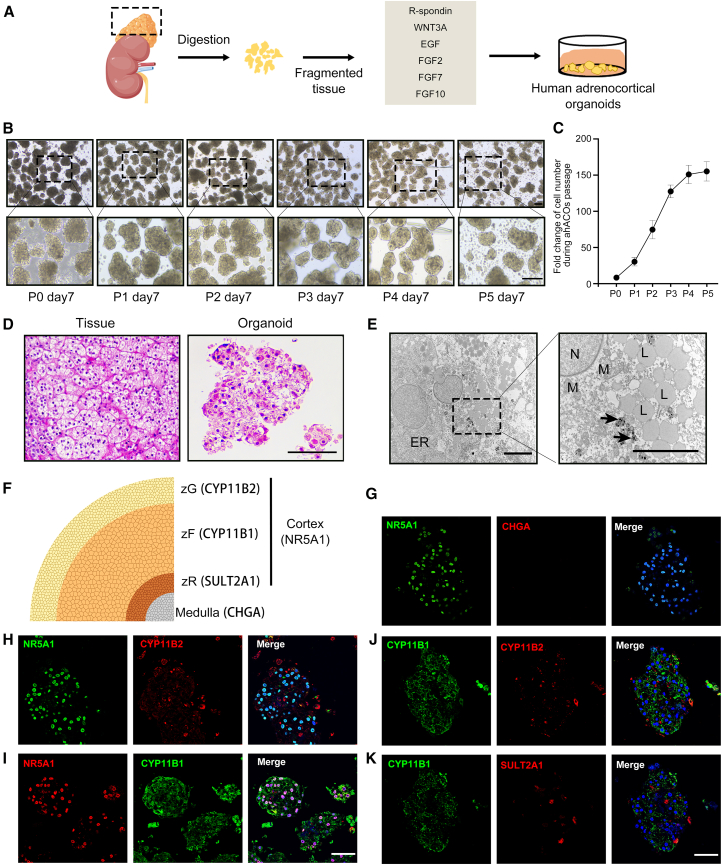
Establishment of an expandable human adrenocortical organoid culture (A) Schematic illustrating the digestion and seeding of adult human adrenal cells in ACO medium. (B) Representative bright-field images of ACOs cultured in ACOs medium at day 7 of passage 0 (P0) to passage 5 (P5) (*n* = 5 donors). Scale bars, 100 μm. The dotted box indicated a zoomed region. (C) Quantification fold change in cell number during ACO passages P0 to P5 (*n* = 5 independent experiments). Each 10,000 primary adrenal cells derived from one patient were mixed with ACO medium and seeded in 24-well low-adhesion plates at day 0 in P0, then cells on day 20 of each passage were counted. Fold change of cell number during passage was defined using the ratio of the cell number after passage to the number of original cells. (D) H&E staining of ACOs and human adrenal cortex tissue. Scale bars, 100 μm. (E) Transmission electron microscopy of ACOs showing typical morphological characteristics resembling adrenal zF. In the magnification, mitochondria (M), lipid droplets (L), and lysosomes (arrow) in cytoplasm were clearly visible. N, nucleus; ER, endoplasmic reticulum. Scale bars, 1 μm. (F) Schematic diagram of cell markers in different regions of the adrenal gland. (G–K) Representative immunofluorescence (IF) images for the indicated markers illustrating cellular composition and organization in expanded ACOs (P2). Nuclei were counterstained with DAPI (blue). IF double-labeled ACOs of NR5A1 and CHGA (G), NR5A1 and CYP11B2 (H), NR5A1 and CYP11B1 (I), CYP11B1 and CYP11B2 (J), CYP11B1 and SULT2A1 (K). Scale bars, 50 μm.

To develop a research model for the human adrenal cortex, a culture system for adult human ACOs was established. Normal adrenal tissues adjacent to non-cortisol-secreting benign adrenal tumors were collected (*n* = 5; Table S1), and normal adrenocortical cells were isolated through mechanical and enzymatic tissue disruption. The adrenocortical cells were cultured in suspension (Figure 1A). To optimize ACO culture conditions, we added the commonly used factors for human organoids (including R-spondin1, epidermal growth factor [EGF], FGF10, and other basic components of organoids culture) and key factors that promote adrenal cortex cell growth (including WNT3A, FGF2, and FGF7) (Kim and Choi, 2020; Sakata et al., 2022) to formulate ACO medium. To assess the importance of maintaining growth and structural integrity, we removed each one from the medium and identified WNT3A and EGF as indispensable for the primary growth of ACOs. Fibroblast growth factors (FGFs) (FGF2, FGF7, and FGF10) were necessary for maintaining structural integrity (Figure S1A). Compared to suspension culture, adrenocortical cells exhibited adherent growth when embedded in Matrigel, with most cells lost during the passage (Figure S1B). The optimized ACO medium sustained rapid growth of ACOs from passage 0 (P0) to passage 3 (P3). After P3, the expansion ability of ACOs decreased significantly, although organoids could still be maintained for more than 3 months without morphological changes (Figures 1B and 1C). Histological analysis revealed that ACOs contained cells with morphological features similar to those of the human adrenal cortex, particularly the cellular features of the zF and zG. However, the overall tissue architecture with its defined zonation was not recapitulated (Figure 1D). Under the electron microscope, the cytoplasm of ACOs contained abundant endoplasmic reticulum, rich round or oval mitochondria, a few numbers of lysosomes, as well as numerous lipid droplets (Figure 1E). The ultrastructural features of these ACO cells were consistent with those of primitive human and rodent adrenal zF (Annamaraju et al., 2021; Johnny et al., 2021). We characterized cell identities of these proliferating ACOs (Figure S1C, P1 and P2) by immunofluorescence (Figures 1F–1K). Organoids mostly comprised NR5A1^+^ cortical cells and had nearly no CHGA^+^ medullary cells (Figure 1G). The co-staining of NR5A1, CYP11B1, and CYP11B2 indicated that the organoids were primarily composed of CYP11B1^+^ zF cells (Figures 1H–1J). The number of SULT2A1^+^ zR cells was rather limited (Figure 1K). Taken together, our culture conditions enabled the fast short-term expansion (within P3) of ACOs and maintained long-term survival (more than 3 months) while retaining major characteristics of adrenal cortical cells.

### Single-cell atlas of human ACOs pinpointed the preservation of functional adrenocortical lineages

**Figure 2:**
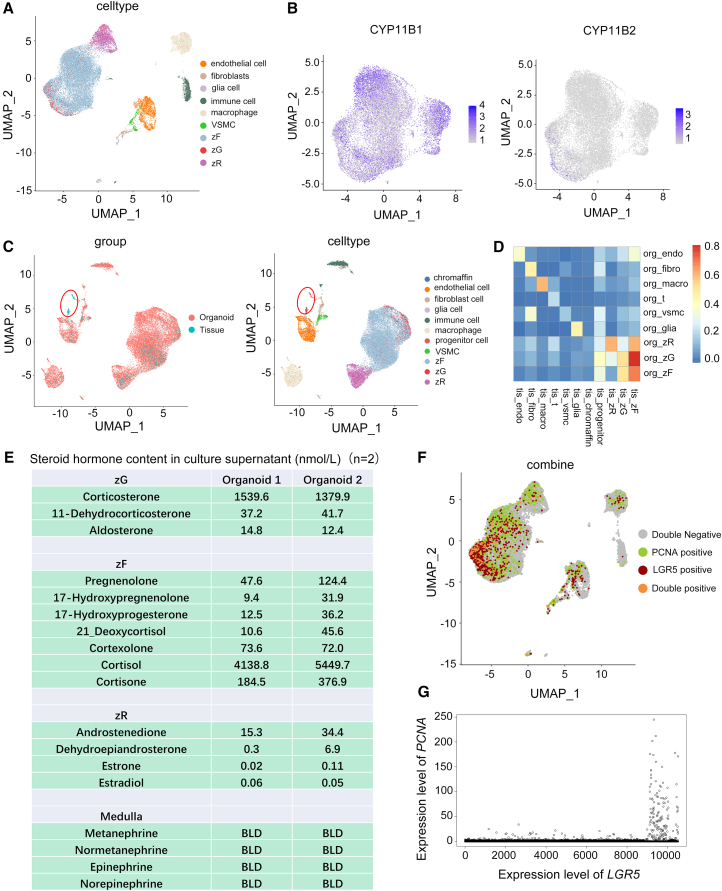
Single-cell atlas analysis in human ACOs reveals the preservation of functional adrenocortical lineages (A) Uniform manifold approximation and projection (UMAP) plot revealing nine main cell type clusters identified in ACOs (*n* = 3 donors). (B) Expression of signature genes of zF (CYP11B1) and zG (CYP11B2) in adrenocortical cells of ACOs (*n* = 3 donors). (C) On the left: UMAP plot of combined ACOs and adrenal glands data colored by sample type. On the right: UMAP plot revealing 11 combined clusters identified from ACO and adrenal gland data (*n* = 3 donors). (D) Correlation plot comparing organoid tissue clustered with assigned cell classes between ACOs (org_) and primary adrenal glands (tis_) (*n* = 3 donors). (E) Steroid hormone content in ACO culture supernatants of two patients (nmol/L, *n* = 2 donors). BLD, below limit of detection. (F) Expression of *LGR5* and *PCNA* in ACOs. (G) Classification of cells according to the expression level of *LGR5*, showing *PCNA* expression in the dot plot (*n* = 3 donors). *x* axis: the expression level of *LGR5*; *y* axis: the expression level of *PCNA*.

To investigate the cellular heterogeneity of the ACOs in more detail, we performed single-cell sequencing of three independent ACOs (day 7 of P2) produced from individual patients. Based on previously reported adrenal cell markers (Bedoya-Reina et al., 2021; Huang et al., 2021), nine main known cell types were identified in ACOs (Figures 2A, S2A, and S2B): zG (high *CYP11B2* expression), zF (high *STAR and CYP11B1* expression), zR (high *CYB5A* expression), endothelial cells, fibroblasts, glia cells, immune cells, macrophages, and vascular smooth muscle cells. zG, zF, and zR cells belonged to the adrenocortical cells, which accounted for roughly 75% of all cells in the organoids (Figure S2C). To interrogate the adrenocortical populations further, we re-clustered cells from clusters zG, zF, and zR at 0.3 resolution and subsequently analyzed the expression patterns of several cortical markers within them. We observed a high expression of *CYP11B1* or *CYB5A* in cells with elevated *STAR* expression, while a few cells expressed *CYP11B2* (Figures 2B and S2D). These results indicate that the gene expression patterns and cell proportions of cortical cells in ACOs are consistent with those in the human adrenal tissues.

To facilitate a direct comparison between organoid and human adrenal gland, we combined our organoid single-cell data with published human adrenal gland single-cell data (Bedoya-Reina et al., 2021; Huang et al., 2021). The combined dataset represented expression information for 42,216 genes within 30,636 cells (including 1,312 cells from four adrenal tissues and 29,324 cells from three ACOs). In contrast to primary adrenal tissues, the organoids lacked two distinct cell clusters: progenitor cells and medullary cells (Figure 2C). We then performed organoid-tissue cluster correlation comparisons using the adult adrenal tissue dataset. It was noted that cortical tissue clusters and ACO clusters with the same annotated cell identity were intermingled in the correlation analysis (Figure 2D). Given that zF cells predominated in both ACOs and adrenal tissue, which mainly synthesized and secreted glucocorticoids such as cortisol and cortisone, we next used mass spectrometry (MS) to analyze the culture supernatants of ACOs. MS revealed the presence of high concentrations of cortisol and cortisone, whereas aldosterone concentrations were low. Catecholamines (epinephrine and norepinephrine) and their metabolites (metanephrine and normetanephrine) were undetectable (Figure 2E). Thus, the hormonal secretion profile corroborated the results of the single-cell expression analysis.

As described earlier, the progenitor cell population was absent in ACOs (Figure 2C). To elucidate mechanisms underpinning ACO proliferation and passage capability, cells were sorted based on the expression level of *LGR5* and assessed for the expression of *PCNA*. LGR5 has been identified as a marker of stem cells in multiple organs (Barker et al., 2007; Huch et al., 2013) and may also be a potential stem cell marker of the adrenal gland (Shaikh et al., 2015), whereas PCNA is a marker of cell proliferation (Wang, 2014). The ACOs contained many *LGR5*^+^/*PCNA*^−^ and *LGR5*^−^/*PCNA*^+^ cells, as well as *LGR5*^+^/*PCNA*^+^ cells (Figures 2F and 2G). In the absence of progenitor cells, these cell populations—comprising *LGR5*^+^/*PCNA*^−^, *LGR5*^−^/*PCNA*^+^, and *LGR5*^+^/*PCNA*^+^ cells—may drive the proliferation of ACOs. To further investigate the relationship between *LGR5* expression and ACO proliferation, ACOs (passage 2) from three independent donors were cultured under three distinct conditions: Adf12 medium (advanced DMEM/F12 basal medium only), ACOM-GFs (ACO culture medium depleted of FGFs, EGF, WNT3A, and R-spondin 1), and ACOM (standard complete ACO culture medium). Following 7 days of culture, organoid colony formation efficiency was assessed, and the relative expression levels of *LGR5* and *PCNA* were quantified, implying that *LGR5* expression was positively correlated with ACO proliferation, colony formation efficiency, and the expression levels of *PCNA* (Figures S2E–S2G).

### Adrenocorticotropic hormone treatment enhanced the maturation and cortisol secretion of human ACOs

**Figure 3:**
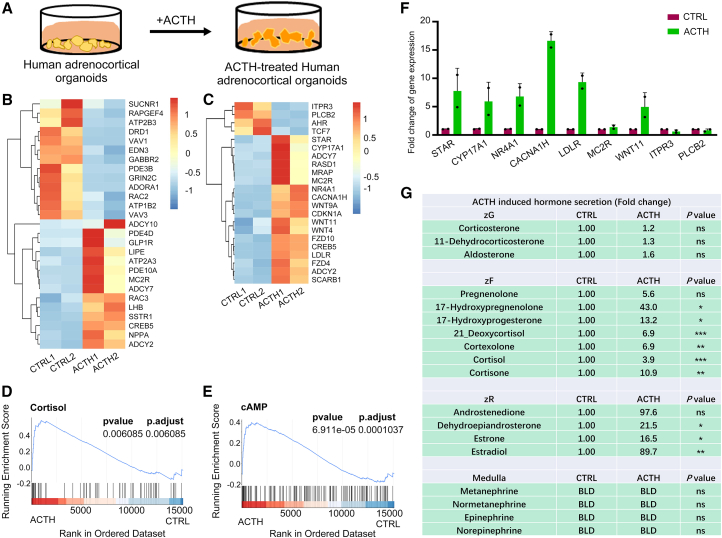
ACTH treatment enhances the maturation and cortisol secretion of human ACOs (A) Schematic diagram of the experimental procedure for ACTH stimulation of ACOs. (B) Heatmap of reported cAMP-related genes in control (CTRL) and ACTH-treated ACOs (*n* = 2 independent experiments). (C) Heatmap of reported cortisol-related genes in CTRL and ACTH-treated ACOs (*n* = 2 independent experiments). (D and E) GSEA analysis showing enrichment of cortisol synthesis (D) and cAMP signaling (E) in ACTH-treated ACOs versus CTRL ACOs (*n* = 2 independent experiments). (F) RT-qPCR analysis of gene expression of cortisol synthesis and cAMP pathway genes (data are presented as mean ± SD of two independent donors, each representing the average of 3 technical replicates). (G) Quantification of hormone content in culture supernatants of CTRL and ACTH-treated ACOs (*n* = 3 independent experiments per group). ^∗^*p* < 0.05; ^∗∗^*p* < 0.01; ^∗∗∗^*p* < 0.001.

Adrenal steroid synthesis is regulated by ACTH. Hence, ACTH_1-39_ was added to the culture medium to determine whether these ACOs were responsive to ACTH (Figure 3A). To understand the ACTH-induced cellular and molecular changes in ACOs, global gene expression profiles of control and ACTH-treated ACOs derived from two independent patients were assessed by RNA sequencing. Using DESeq2, 1,244 differentially expressed genes (DEGs) were identified between control and ACTH-treated ACOs, including 488 upregulated and 756 downregulated genes (Figure S3A). It is prominent that Kyoto Encyclopedia of Genes and Genomes (KEGG) and Gene Ontology (GO) analyses revealed significant enrichment of DEGs in steroid biosynthetic and metabolic pathways (Figures S3A and S3B). We further observed the upregulated expression of genes associated with cAMP signaling (Figure 3B) and steroid hormone synthesis (Figure 3C) in ACTH-treated ACOs. Consistently, gene set enrichment analysis (GSEA) of DEGs confirmed upregulation of cortisol generation and cAMP signaling in ACTH-treated ACOs (Figures 3D and 3E). In addition, a few selected crucial DEGs were also verified by quantitative reverse-transcription PCR (RT-qPCR) (Figure 3F).

The MS analysis of the culture supernatants showed a significant increase of glucocorticoids (cortisol and cortisone) and their precursors in ACTH-treated ACOs compared to controls, whereas mineralocorticoids and catecholamines were low or only slightly affected by ACTH (Figures 3F and S3C). Additionally, we also treated ACOs with angiotensin II. As expected, angiotensin II treatment significantly enhanced the ability of ACOs to secrete aldosterone and also promoted the secretion of certain steroid hormones in zF and zR (Table S4). These results indicate that ACOs are responsive to both ACTH and angiotensin II stimulation.

### Human ACOs exhibited tissue regeneration and cortisol secretion *in vivo* upon subcutaneous transplantation

**Figure 4:**
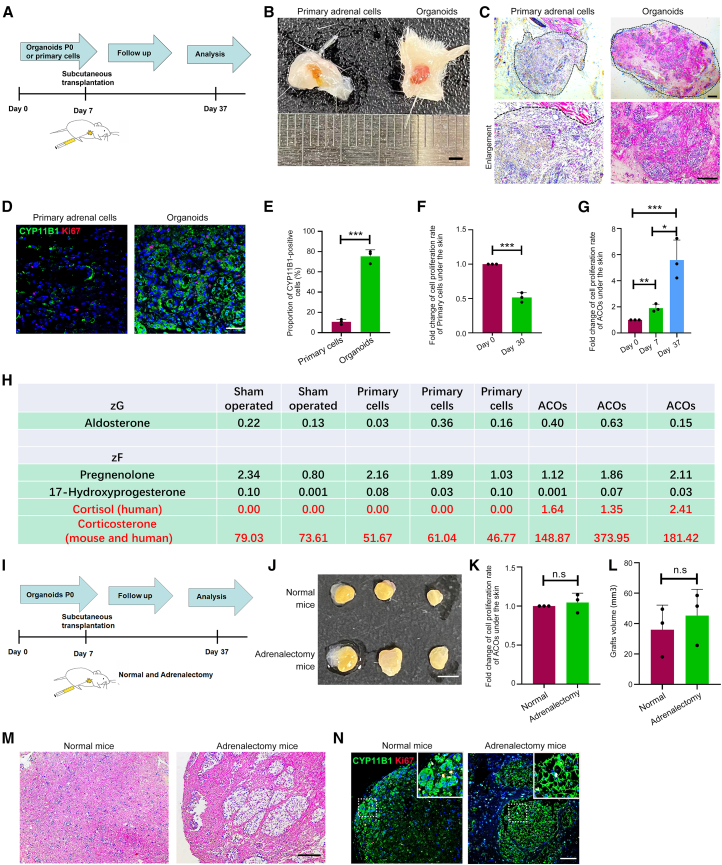
Human ACOs exhibit tissue regeneration and cortisol secretion *in vivo* upon subcutaneous transplantation (A–G) The adrenal primary cells and derived ACOs of four patients were transplanted subcutaneously into NSG mice (*n* = 4 donors for each group), and xenografts and serum were collected. (A) Schematic of the *in vivo* transplantation experiments. (B) Representative photograph of the skin of NSG mice with primary adrenal cells and ACOs transplanted subcutaneously. Scale bars, 2 mm. (C) Representative H&E staining of xenografts derived from primary adrenal cells and ACOs. Black dashed curves outline the ACO xenograft. Scale bars, 100 μm. (D) Representative IF staining of CYP11B1 (green), KI67 (red), and DAPI (blue) in graft tissues. Scale bars, 25 μm. (E) Comparison of the percentage of CYP11B1^+^ cells in transplanted xenograft derived from primary adrenal cells and ACOs (*n* = 3 donors for each group). ^∗∗∗^*p* < 0.001. (F) Proliferation of adrenal primary cells decreased significantly after transplantation (*n* = 3 donors for each group). ^∗∗∗^*p* < 0.001. (G) Proliferation of ACOs increased significantly after transplantation (*n* = 3 donors for each group). ^∗^*p* < 0.05; ^∗∗^*p* < 0.01; ^∗∗∗^*p* < 0.001. (H) Serum steroid hormone content in sham-operated control mice (*n* = 2 donors) and mice with subcutaneous transplantation of adrenal primary cells and ACOs (ng/mL, *n* = 3 donors for each group). (I) Schematic of the *in vivo* transplantation experiments to compare the effects of ACO transplantation subcutaneously in normal and adrenalectomized mice. (J) Photograph of ACO xenografts derived from normal and adrenalectomized mice (*n* = 3 donors for each group) after day 30 of transplantation. Scale bars, 3 mm. (K) Proliferation of ACOs under the adrenalectomized mice skin was similar to that under the normal mice skin (*n* = 3 donors for each group). (L and M) Volume measurement (L) and H&E staining analysis (M) of ACO xenografts derived from normal and adrenalectomized mice 30 days after transplantation (*n* = 3 donors for each group). Scale bars, 100 μm. (N) Representative IF staining of CYP11B1 (green), KI67 (red), and DAPI (blue) in ACO graft tissues derived from normal and adrenalectomized mice after day 30 transplantation (*n* = 3 donors for each group). Scale bars, 50 μm. Insets were higher magnifications of the corresponding regions indicated by the dashed boxes.

Most previous studies employed primary adrenal cells derived from large mammals or humans for transplantation into rodents (Balyura et al., 2015; Maria et al., 2023; Tanaka et al., 2020; Thomas et al., 2000). To demonstrate the advantage of ACO transplantation in regenerating the adrenal function *in vivo* compared to human primary adrenal cells (not cultured into organoids), ACOs and primary adrenal cells were transplanted under the skin of NSG mice, respectively (Figure 4A). One month later, ACOs formed larger and more rounded grafts compared to primary cells (Figure 4B). H&E staining showed that grafts formed by ACOs comprised more zF cells and exhibited a more complete structure. In contrast, very few zF cells but more macrophages were observed in the graft formed by the primary cells (Figure 4C). Compared with the primary cells, ACO-derived grafts possessed more CYP11B1^+^ cells (Figures 4D and 4E) and Ki67^+^-proliferative cells (Figure 4D). These data showed that ACOs outperformed primary cells with improved ability to sustain transplantation. We next analyzed the cell number of primary cells and ACOs before and after transplantation and found that the cell number of primary cells significantly decreased after subcutaneous growth for 30 days, while the cell number of ACOs showed a marked increase under the same condition (Figures 4F and 4G). Further examination of serum steroid hormones in mice subcutaneously transplanted with human ACOs (*n* = 3) illustrated that the transplanted ACOs were capable of secreting a small amount of cortisol (source: human) and corticosterone (source: human and mouse) *in vivo* compared to sham-operated mice (*n* = 2) and mice subcutaneously transplanted with human adrenal primary cells (*n* = 3) (Figure 4H).

### Human ACOs restored glucocorticoids homeostasis to rescue adrenalectomized mice

To explore the proliferation and cortisol secretion of ACOs under the skin of adrenalectomized mice, we transplanted the same number of organoids subcutaneously into NSG mice with or without adrenalectomy (4 vs. 4), respectively. After 30 days, grafts and serum were collected (Figure 4I). As shown in Figures 4J–4L, there was no difference in the cell number and the size of grafts between the two groups. The histological characteristics and the expression pattern of CYP11B1 of the grafts generated subcutaneously in adrenalectomized mice resembled human zF also with regard to secretory function (Figures 4M, 4N, S4A, and S4B).

**Figure 5:**
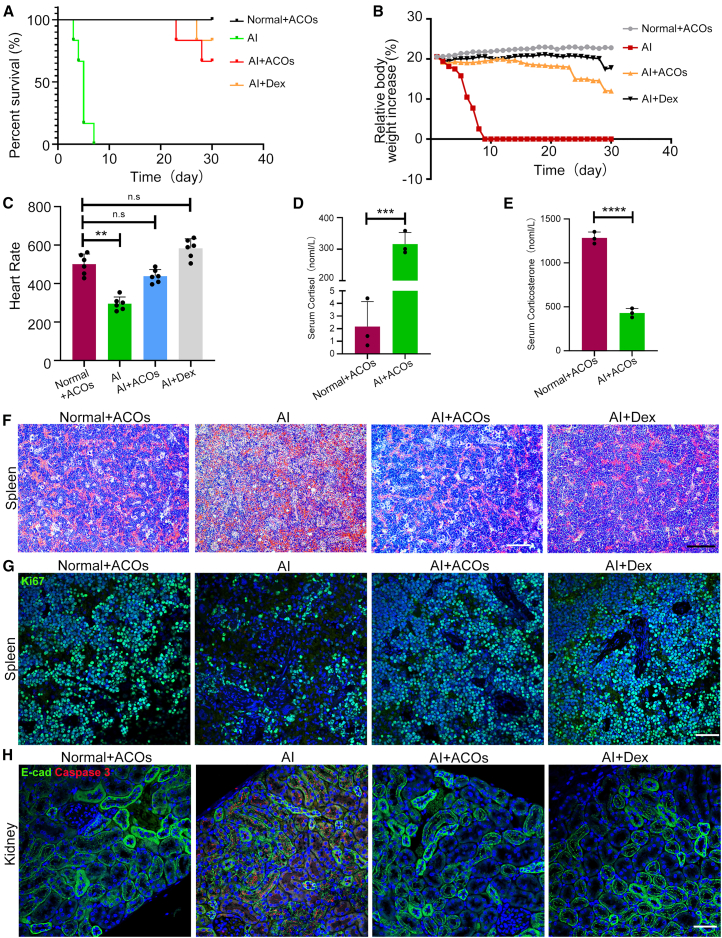
Human ACOs restore glucocorticoid homeostasis to rescue adrenalectomized mice (A) Survival curve of Normal + ACOs, AI, AI + ACOs, and AI + Dex mice (*n* = 6 independent experiments for each group). Normal + ACOs, normal healthy mice receiving subcutaneous ACO transplantation; AI, adrenal insufficient mice induced by bilateral total adrenalectomy; AI + ACOs, adrenalectomized mice receiving subcutaneous ACOs transplantation, and AI + Dex, adrenalectomized mice receiving daily intraperitoneal dexamethasone supplementation. (B) Body weight monitoring of Normal + ACOs, AI, AI + ACOs, and AI + Dex mice (*n* = 6 independent experiments for each group). (C) Heart rate of mice (*n* = 6 independent experiments for each group), at 4 days after adrenalectomy or transplantation (^∗∗^*p <* 0.01). (D and E) Serum levels of cortisol (D) and corticosterone (E) in Normal + ACOs and AI + ACOs mice 30 days post transplantation (nmol/L, *n* = 3 independent experiments for each group). ^∗∗∗^*p* < 0.001; ^∗∗∗∗^*p* < 0.0001. (F) H&E staining of mouse spleen at 6 days after adrenalectomy or transplantation (*n* = 2 independent experiments). Scale bars, 100 μm. (G) IF staining of Ki67 (green) and DAPI (blue) in spleen tissue of Normal + ACOs, AI, AI + ACOs, and AI + Dex mice (*n* = 2 independent experiments). Scale bars, 25 μm. (H) IF staining of caspase-3 (red) and DAPI (blue) in kidney tissue of Normal + ACOs, AI, AI + ACOs, and AI + Dex mice (*n* = 2 independent experiments). Scale bars, 25 μm.

To assess the ability of human ACOs to rescue adrenal insufficiency, mice were divided into four groups (*n* = 8 per group): (1) normal healthy mice receiving subcutaneous ACO transplantation (Normal+ACOs), (2) adrenal-insufficient mice induced by bilateral total adrenalectomy (AI), (3) adrenalectomized mice receiving subcutaneous ACO transplantation (AI + ACOs), and (4) adrenalectomized mice receiving daily dexamethasone supplementation (AI + Dex). Two mice from each group were sacrificed, and organs were collected for comparative analysis on day 6. The other six mice in each group were monitored for 30 days to assess survival. As shown in Figure 5A, adrenalectomized mice without treatment died within 9 days after adrenalectomy, consistent with previous studies (Michael et al., 1997; Thomas and Hornsby, 1999). These mice exhibited severe symptoms of adrenal insufficiency, with progressive weight loss (Figures 5B and S5A), decreased motor activity, and apathy. All adrenalectomized animals receiving ACO transplantation and dexamethasone were continuously monitored for 30 days. Their body weight increased after a slight decline during the initial 3 days (Figure 5B). For the first 20 days, these mice remained active without symptoms of apathy. Nevertheless, from day 15 onward, the weight of adrenalectomized mice with ACOs started to decline (Figure 5B). One mouse died on day 23 and another died on day 28 after transplantation. One adrenalectomized mouse with dexamethasone supplementation also died on day 27 (Figure 5A). The heart rate was normalized by ACO xenograft and dexamethasone treatment (Figure 5C). Serum cortisol levels dramatically increased (Figure 5D), while serum corticosterone levels significantly decreased in adrenalectomized mice with ACOs, compared to normal mice with ACOs (Figure 5E). These data suggest that early-passage ACOs could rescue adrenalectomized mice by producing cortisol and corticosterone.

To investigate whether later-passage ACOs are able to rescue adrenalectomy, we performed subcutaneous transplantation in adrenalectomized NSG mice using P2 organoids (passaged twice, 21 days total *in vitro* culture). Mice were divided into four groups (*n* = 6 per group): (1) sham-operated group (Sham), (2) adrenal-insufficient mice by bilateral total adrenalectomy (AI), (3) adrenalectomized mice receiving subcutaneous ACO transplantation (AI + ACOs), and (4) adrenalectomized mice receiving daily dexamethasone supplementation (AI + Dex). In the AI group, five mice succumbed within 5 days post adrenalectomy, whereas one mouse survived throughout the 30-day observation period. This might be attributed to incomplete resection of adrenal glands. In contrast, among mice transplanted with P2 organoids, only one death occurred, and the surviving individuals exhibited gradual weight gain over the 30-day period (Figures S5B and S5C). These results demonstrate that later-passage ACOs can also rescue adrenalectomy.

To evaluate the physiological impact of early-passage ACOs, two mice in each group were sacrificed 6 days post transplantation. H&E staining and immunofluorescence (IF) analysis were performed on representative target organs of glucocorticoids including the spleen, kidney, liver, and intestine. Compared to the normal mice with ACOs, the quantity of proliferating lymphocytes within the spleen of the adrenalectomized mice was markedly reduced (Figure 5F), and the proportion of Ki67^+^ cells was notably lower (Figure 5G). The ACO transplantation and dexamethasone injection rescued the significant decrease of proliferating lymphocytes in the spleen. Although we did not identify abnormal changes in the H&E staining of the kidneys (Figure S5D), a considerable increase in the staining of cleaved caspase-3 (a marker of apoptotic cells) was observed in the various tubules of the kidneys in the adrenalectomized mice, which was rescued by ACO transplantation and dexamethasone, respectively (Figure 5H). No significant changes were observed in the liver or intestine on H&E staining. These findings are consistent with the critical role of ACOs in maintaining homeostasis of adrenalectomized mice.

### The application of human ACOs in adrenal disease modeling

**Figure 6:**
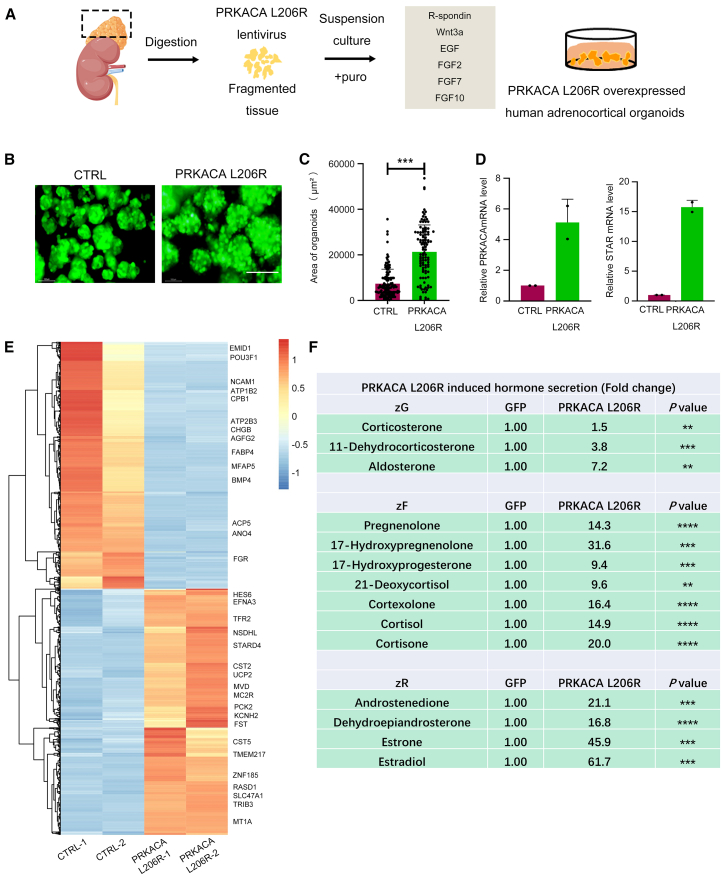
Introducing PRKACA L206R into human ACOs models the disease of cortisol-producing adenomas (A) Principle of tumor modeling in ACOs using lentiviral transfection. ACOs from the same patient were infected with GFP and PRKACA L206R viruses, respectively. (B) Representative GFP fluorescence images of control (CTRL; GFP lentivirus transfection) and PRKACA L206R ACOs (P2) after puromycin selection (*n* = 3 donors per group). Scale bars, 100 μm. (C) Quantification of organoids area in CTRL and PRKACA L206R ACOs (*n* = 128 organoids for CTRL group; *n* = 105 organoids for PRKACA L206R group; 3 different donors were used for each group). ^∗∗∗^*p* < 0.001. (D) RT-qPCR analysis of *PRKACA* and *STAR* expression (data are presented as mean ± SD of two independent donors, each representing the average of 3 technical replicates). (E) Heatmap of DEGs between CTRL ACOs and PRKACA L206R ACOs (*n* = 2 donors per group) (*p <* 0.05 and |log2 fold change|≥1.5). Signature genes associated with ACA were marked out. (F) Quantification of hormone content in supernatants of CTRL and PRKACA L206R ACOs (*n* = 3 donors per group). ^∗∗^*p <* 0.01; ^∗∗∗^*p <* 0.001; ^∗∗∗∗^*p <* 0.0001.

To investigate whether ACOs allowed disease modeling by genetic manipulation, we focused on cortisol-producing adrenocortical adenoma (ACA). A hotspot somatic PV (L206R) in PRKACA is the most frequent cause of cortisol-producing ACAs, which leads to Cushing’s syndrome (Sato et al., 2014; Yanan et al., 2014). However, there is no mouse model for ACA harboring the PRKACA L206R PV (Sato et al., 2014). Therefore, we introduced the PRKACA L206R PV into the ACOs via lentiviral transfection (Figure 6A). Overexpression of PRKACA L206R (GFP and qPCR shown in Figures 6B and 6D) significantly promoted the growth of the ACOs (Figures 6B and 6C) and upregulated the expression of *STAR* gene involved in steroid synthesis (Figure 6D). To validate that the PRKACA L206R-overexpressing ACOs obtained the key features of ACA, transformed organoids were subjected to transcriptome analysis, revealing 754 upregulated and 891 downregulated DEGs (*p* < 0.05 and |log2 fold change|≥1.5). Particularly, PRKACA L206R PV induced the expression of many signature genes of ACA, including *HES6*, *STARD4*, *CST5*, *SLC47A1*, *CHGB*, and *BMP4* (Figure 6E). The expression patterns of these signature genes were consistent with the reported ACA transcriptome (Sato et al., 2014). GSEA, GO, and KEGG analyses revealed that PRKACA L206R organoids significantly increased the expression of steroid- and Cushing-associated signature genes (Figures S6A–S6C). MS analysis showed that the steroid hormones secreted by PRKACA L206R organoids were significantly increased (Figures 6F and S6D). These data indicate that the PRKACA L206R PV transformed normal ACOs to ACA-like organoids.

Remarkably, DEGs from PRKACA L206R organoids were significantly enriched in several pathways associated with tumorigenesis and tumor invasion, such as phosphatidylinositol 3-kinase-AKT, Rap1, extracellular matrix receptor, cell adhesion, and mitogen-activated protein kinase cascade, which have not been reported in previous ACA studies (Sato et al., 2014; Yanan et al., 2014; Zennaro et al., 2017). Thus, by utilizing the human adrenal cortex organoid model, we uncovered several tumor-driving pathways in the ACA process, providing a potential mechanistic framework for the occurrence and development of ACA as well as future treatment (Figure S6C).

## Discussion

Organoid cultures can be established from pluripotent stem cells and adult stem cells (Schutgens and Clevers, 2020). To date, studies of endocrine organoids have mainly focused on pancreatic islets and thyroid, both of which are derived from endoderm and have abundant adult stem cells highly capable of proliferation (Jeon et al., 2023). In contrast, the adrenal cortex originates from the mesoderm and primarily consists of functional cells capable to produce steroids, with limited pluripotency. A recent study reconstituted human adrenocortical specification and steroidogenesis using iPSCs, producing fetal zone adrenal-cortex-like cells (Sakata et al., 2022). Nevertheless, these cells mainly produce dehydroepiandrosterone-sulfate (DHEA-S), whereas adult adrenal cells chiefly secrete cortisol and aldosterone. Consistently, in an early study, transplantation of fetal human adrenal cells, which mainly produced DHEA-S, could not rescue adrenalectomized mice (Popnikolov and Hornsby, 1999). So far, there have been no successful human normal adrenal cortex or medulla organoid cultures.

Transplantation of a cell clone derived from primary bovine adrenal cortical cells in adrenalectomized SCID mice was attempted as early as in 1997 (Thomas et al., 2000). In the original study, the clone of cells was transplanted beneath the kidney capsule in a polycarbonate cylinder with FGF1-secreting 3T3 cells to support its growth. Several subsequent studies have tested similar approaches with modifications and concluded that primary bovine adrenal cells and primary human adrenal cells can also replace murine adrenal function, while fetal adrenal cells cannot; and the co-transplantation with FGF1-secreting 3T3 cells is essential for the revascularization and survival of the graft (Popnikolov and Hornsby, 1999; Thomas and Hornsby, 1999; Thomas et al., 2002). After that, the transplantation of primary bovine adrenocortical cells encapsulated in alginate was also proved to be successful in reversing induced adrenal insufficiency in rodents (Balyura et al., 2015). Transplantation of cultured primary porcine adrenal spheroids beneath the kidney capsule also rescued mice following adrenalectomy (Maria et al., 2023). Recently, autologous porcine adrenal cortical cell transplantation was attempted, though without clinically relevant hormone production (Clarke et al., 2024). In this study, we demonstrate that *ex vivo*-expanded human adrenal organoids can help adrenalectomized mice to survive. Two mice receiving ACOs from older donors died after transplantation, suggesting that the viability of ACOs might be affected by donor heterogeneity based on factors like age, sex, etc. A recent proof-of-concept study showed that allogeneic pancreatic beta cells, with *B2M* (encoding a component of class I human leukocyte antigen [HLA]) and *CIITA* (encoding a master regulator of class II HLA transcription) genes inactivated by Cas12b and *CD47* overexpressed by a lentiviral vector, survived without immunosuppression, when transplanted into the forearm muscle. We believe that allogeneic ACOs could be manipulated in a similar approach to avoid rejection, before transplanted into the forearm muscle or into the liver via the portal vein (Carlsson et al., 2025). Nevertheless, the present study still represents a significant advancement toward a better solution for human primary adrenal insufficiency.

Combined with genetic manipulation, like introducing a hotspot PV of PRKACA via lentiviral transfection to mimic cortisol-producing adenomas, these organoids could serve as an ideal tool for drug screening. In 2020, osilodrostat, which decreases cortisol by inhibiting 11-β hydroxylase, was approved by the Food and Drug Administration for the treatment of Cushing’s syndrome (Duggan, 2020). It would be intriguing to explore and identify novel compounds that could precisely inhibit the production of cortisol in adenomas with PRKACA PV, while sparing normal adrenals in future studies. This proof-of-concept study demonstrates the great potential of these organoids as a valuable tool for studying adrenal cortex disorders.

However, the present study has several limitations. Firstly, the ACOs were able to self-assemble and grow only in floating culture instead of in Matrigel. The limited proliferative capacity of adult adrenal stem cells has made it very difficult to grow organoids from single cells in Matrigel. Secondly, without a medulla core, these ACOs differ from normal adrenal cortex, which undergoes constant renewal toward the core following a centripetal pattern. It remains to be optimized in the future so that these ACOs will adopt a more physiological behavior as human adrenal glands. Thirdly, these ACOs were predominantly composed of cells resembling zF; it remains to be tested whether they were suitable for studying disorders related to zG, e.g., primary aldosteronism. Finally, although we optimized culture protocols across multiple donors, the inherent proliferative barrier of adult ACOs prevented detailed longitudinal analysis of zone-specific marker trajectories (e.g., zG/zF/zR/stem cell genes) and niche remodeling during passaging. Future work should integrate multi-donor sampling at every passage for single-cell RNA sequencing and qPCR profiling to systematically resolve cellular dynamics and transcriptional adaptations in expanding ACOs.

In conclusion, our study has established a short-term passaged adrenal cortex organoid culture system using normal human adrenal glands. These organoids mainly consist of adrenocortical cells and produce cortisol in a regulated manner, which is a characteristic of the zF. Combined with genetic manipulation, these organoids will serve as a critical foundation for studying human adrenal cortex diseases, as exemplified by introducing PRKACA PV to mimic Cushing’s syndrome. Furthermore, these organoids will also serve as a stepping stone for adrenal regeneration and demonstrate the potential of organoid transplantation to restore adrenal function at the physiological level. Therefore, our organoid model provides a powerful and promising approach to restore endocrine regulation in patients suffering from adrenal insufficiency.

## Methods

### More information in supplemental methods

#### Ethical regulation compliance

The research was conducted in accordance with the Declaration of Helsinki, and the use of human tissues for this study was approved in accordance with the Declaration of Helsinki and Good Clinical Practice guidelines. All experiments with adult adrenal gland tissue were approved by the Ethical Committee of Zhongshan Hospital of Fudan University (B2023-190) in accordance with all relevant ethical regulations. Animal studies were approved by the Institutional Animal Care and Use Committee at Nanchang University (#10083).

#### Tissue collection and organoid culture

Adult adrenal tissues were stored at 4°C in advanced DMEM/F-12 medium with penicillin-streptomycin (Gibco) until use. Tissues were minced into 2–5 mm^3^ pieces, digested with 500 μL pre-warmed 1,000 U/mL collagenase I(Thermo Fisher Scientific) at 37°C for 30 min, neutralized with 10% FBS, and filtered through a 70 μm strainer. Primary adrenal cells (30,000 cells per well) were mixed with ACO medium and seeded in a 24-well low-adhesion plate and then transferred to humidified 37°C/5% CO_2_ incubators at ambient O_2_. To prepare low-adhesion 24-well plates, a 1.6% Pluronic F-127 (Sigma, P2443) solution was made in PBS. It was added to each well for even coating and was incubated at room temperature for 2 h. The F-127 solution was then removed, and the wells were washed twice with fresh PBS. The plate was subsequently ready for cell seeding. The medium was replaced by fresh ACO medium every 3 days for up to 10 days. ACO medium: advanced DMEM/F-12 medium supplemented with basal medium plus B27 (1×, Gibco), Penicillin-Streptomycin (1%, Procell), 100 μg/mL Primocin (InvivoGen), GlutaMax (1×, Gibco), 10 mM HEPES (Gibco), 1.25 mM N-acetylcysteine (Sigma), 10 mM nicotinamide (Sigma), 500 ng/mL R-spondin1 (bioGenous), 200 ng/mL WNT3A (bioGenous), 50 ng/mL EGF (bioGenous), 100 ng/mL FGF10 (bioGenous), 25 ng/mL FGF7 (bioGenous), 50 ng/mL FGF2 (bioGenous), 500 nM A8301 (Selleck), and 5 μM Rho inhibitor Y-27632 (bioGenous).

#### Single-cell RNA sequencing and data preprocessing

(1) 10× genomics library preparation: dissociated processes of single cells from adult ACOs (passage 2) were mentioned earlier. Single-cell sequencing was performed using the 10× Genomics platform. The 10× Genomics libraries were sequenced as 150-bp paired-end reads on the Illumina HiSeq 4000 platform. Sample demultiplexing, barcode processing, and UMI counting were carried out using the Cell Ranger Software Suite (10× Genomics Cell Ranger 8.0.1), “refdata-gex-GRCh38-2020-A,” was used as reference to map reads on the human genome (GRCh38/hg38). (2) For seurat workflow: raw matrices were loaded into R to conduct subsequent analysis in Seurat v.4.3.0. To get rid of poor-quality cells, clusters where 90% of cells had UMIs less than 500 or a mitochondrial ratio exceeding 20% were filtered out. Genes expressed in less than 10 cells were also filtered. The remaining gene expression matrix was then normalized and scaled, followed by principal-component analysis, CCA integration, and clustering analysis. DotPlot, DimPlot, and FeaturePlot functions in Seurat were used to visualize cell type distribution and specific gene expression patterns. Cells were sorted based on the expression level of *LGR5*, and expression level of *PCNA* was showed alongside *LGR5* in a dot plot. (3) For Spearman correlation analysis: the expression data were divided into subsets corresponding to different cell types within the organoid and tissue. The mean expressions were calculated to represent the expression levels of their corresponding subsets. Finally, the Spearman correlation test was carried out between each of them and projected into a correlation matrix.

#### Animal experiments

(1) For cell transplantation in fibrin clot: thrombin and fibrinogen were obtained from Sigma-Aldrich. Organoids (P0 and P2) and primary cells were mixed with 10 μL of thrombin (10 μ/mL). 10 μL of fibrinogen (10 mg/mL) was added to the cell/thrombin mixture and immediately placed in the pouch under skin of NSG mice. (2) For mouse xenograft model: 6-week NSG (NOD-Prkdcscid Il2rgem1/Smoc) female mice were brought from Shanghai Model Organisms. Subcutaneous cell transplantation was performed by making an incision on the skin of the underarm of the mice using fine scissors, and the ACOs in the fibrin clot were inserted into the incision. Animals were monitored for up to 1 month before xenografts were removed and collected for histology, immunostaining, and cell counting analysis; serum were collected for steroid hormone analysis. (3) For adrenalectomy and transplantation: mice were anesthetized by ketamine/xylazine (100 mg/kg ketamine und 10 mg/kg xylazine). A longitudinal incision was made with fine scissors in the dorsal skin of the retrocostal area. A 1-cm incision in each lateral body wall was made to open the retroperitoneal space. The adrenal glands were ligated and removed. The experimental mice (*n* = 24) underwent adrenalectomy and transplantation in a single surgery. Experimental animals were divided into three groups and received transplantation of 1 × 10^6^ cells: (1) adrenalectomy group (AI, *n* = 8), (2) adrenalectomy combined with subcutaneous organoid transplantation group (AI + ACOs, *n* = 8), and (3) adrenalectomy combined with continuous injection of the dexamethasone group (AI + Dex, *n* = 8). Normal NSG mice with organoid transplantation served as negative controls (Normal + ACOs, *n* = 8). In the Normal + ACOs and AI + ACOs group, a total of eight donors were recruited. The organoids from each donor were evenly divided into two portions, which were respectively transplanted into one mouse in the Normal + ACOs group and one mouse in the AI + ACOs group. Cells from different donors were not mixed before transplantation. Two mice from each group were sacrificed, and organs were collected for comparative analysis on day 6. The other six mice in each group were monitored for 30 days to assess survival and body weight change.

#### Statistical analysis

Values for all quantifications, including RT-qPCR, morphological analysis, cell number, and hormone content, were represented as mean ± SEM. Student’s *t* test was used to determine differences between groups. *p* values for significance were set to 0.05.

## Resource availability

### Lead contact

Further information and requests for resources and reagents should be directed to and will be fulfilled by the lead contact, Bing Zhao (bingzhao@ncu.edu.cn).

### Materials availability

All unique/stable reagents/plasmids/organoids generated in the current study are available from the lead contact on reasonable request.

### Data and code availability

• All sequencing data of this study have been deposited at NODE database (NODE: OEP00006263) and are publicly available.
• All original code has been deposited at GitHub and is publicly available at https://github.com/ShepherdZh/adrenalineSC.
• Any additional information required to reanalyze the data reported in this study is available from the lead contact upon request.

## Acknowledgments

This work was supported by grants from the 10.13039/501100012166National Key Research and Development Program of China (2025YFC3408900 and 2024YFA1802700), the 10.13039/501100001809National Natural Science Foundation of China (82372663 and 82270828), the 10.13039/501100013064Key Research and Development Program of Jiangxi Province (20232BBG70024), and the Key Research and Development Program of Yunnan Province (202302AA310024).

## Author contributions

Q.L., J.J., and B.Z. conceived the study; Q.L. and Xiaoyu Li performed the organoid cultures, transplantation, and staining experiments; Y.Z. performed the bioinformatics; Q.L. analyzed the data; Y.S., Z.L., W.C., and Y.L. collected clinical samples; S.W., X.G., Xuewen Li, and N.B. contributed to the discussion of the work; B.Z. and J.J. supervised the work; and Q.L., J.J., and B.Z. wrote the manuscript.

## Declaration of interests

The authors declare no competing interests.

## Supplemental information

Document S1. Figures S1–S6, Tables S1–S4, and Methods S1

Document S2. Article plus supplemental information
