# Supplementary material for: Human adrenocortical organoids for tissue regeneration and disease modeling
Source: Stem Cell Reports. 2025 Oct 16;20(11):102679. doi: 10.1016/j.stemcr.2025.102679 (PMC12790718; doi:10.1016/j.stemcr.2025.102679)
Supplement: Document S1. Figures S1–S6, Tables S1–S4, and Methods S1 [file mmc1.pdf]

Stem Cell Reports, Volume *20*

Supplemental Information

Human adrenocortical organoids for tissue regeneration and disease modeling

Qing Li, Xiaoyu Li, Yiming Zhang, Yanting Shen, Zhiqiang Lu, Wei Chen, Yujun Liu, Shuang Wu, Xiaofeng Gong, Xuewen Li, Nicole Bechmann, Jingjing Jiang, and Bing Zhao

Supplementary Information

Human Adrenocortical Organoids for Tissue Regeneration and Disease Modeling

Li et al.

Supplementary Figure 1-6

Supplementary Table 1-4

Supplementary methods

Supplementary Figure 1

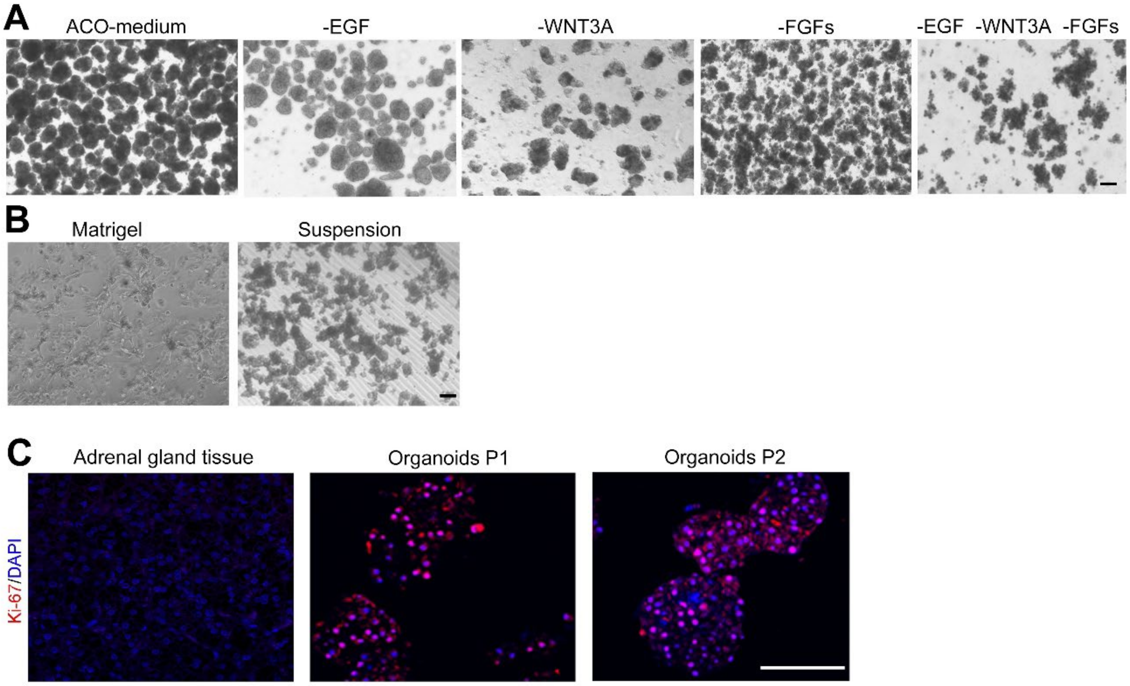

Supplementary Figure 1 | Establishment of an expandable human adrenocortical organoid culture. (**A**) Representative brightfield images of organoids that were cultured in elimination of indicated factors (n=3 independent experiments). Scale bar =100 µm. (**B**) Representative brightfield images of organoids under different culture conditions (Matrigel or suspension culture) (n=3 independent experiments). Scale bar=100 µm. (**C**) Immunofluorescence staining of Ki67 in adrenal tissues and organoids (Passage 1 and 2). Scale bar=50 µm.

Supplementary Figure 2

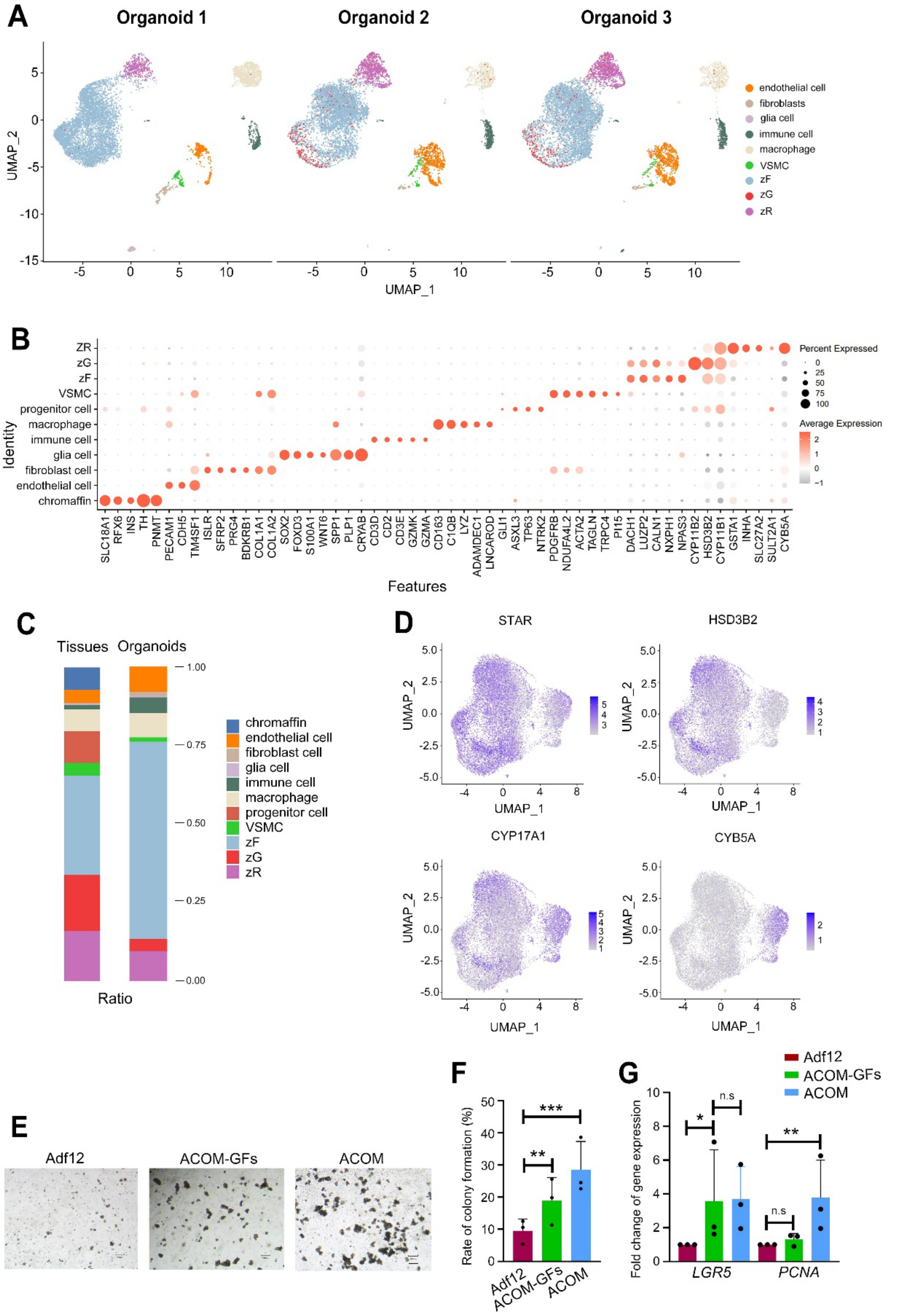

Supplementary Figure 2 | Single-cell atlas analysis in human ACOs reveals the preservation of functional adrenocortical lineages. (**A**) UMAP visualization of the three ACOs (n=3 donors). (**B**) Expression patterns of canonical markers and differentially expressed genes (DEGs) of each cell type. Each dot represents a gene, of which the color saturation indicated the average expression level, and the size indicated the percentage of cells expressing the gene. (**C**) Comparison of general cell type ratio between ACOs and AGs samples (n=3 donors). (**D**) UMAPs of *STAR*, *HSD3B2, CYP17A1* and *CYB5A* expression in adrenocortical cells of ACOs (n=3 donors). (**E-F**) The relationship between *LGR5* expression and ACO proliferation (n=3 independent experiments). (**E**) Bright field images at day 7 of passage 2 ACOs cultured in different medium. (**F**) Quantification of ACO colony formation rate in different culture medium. (**G**) Relative expression of *LGR5* and *PCNA* analyzed by qRT-PCR in ACOs cultured in different medium. Adf12: Advanced DMEM/F12 basal medium only. ACOM-GFs: ACO culture medium depleted of FGFs, EGF, WNT3A, and Rspondin1. ACOM: Complete ACO culture medium. *$p<0.05$; **$p<0.01$; ***$p<0.001$; ****$p<0.0001$.

Supplementary Figure 3

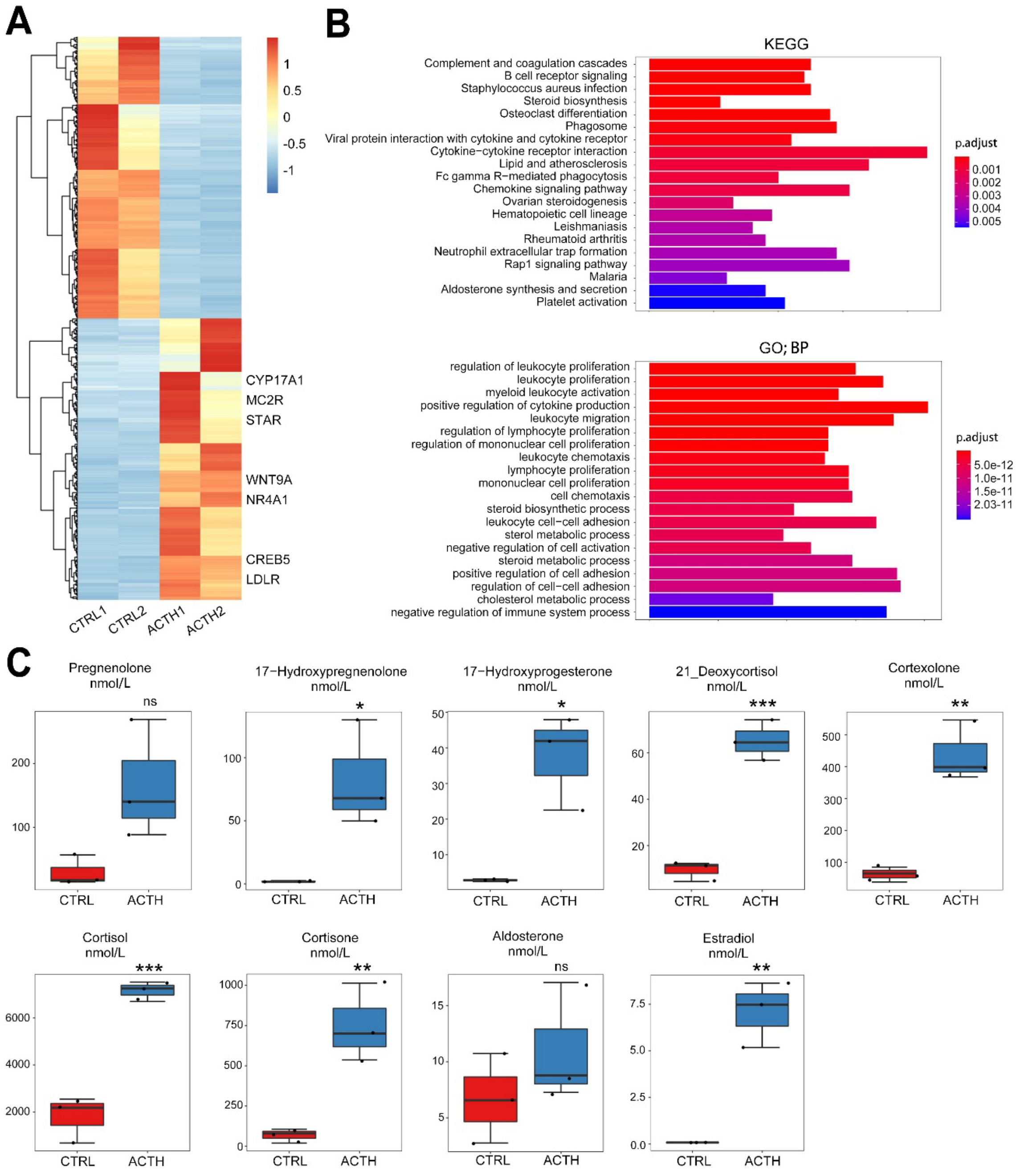

Supplementary Figure 3 | ACTH treatment enhances the maturation and cortisol secretion of human ACOs. (**A**) Heatmap of differentially expressed genes between control ACOs (CTRL) and ACTH-treated ACOs (ACTH) (n=2 independent experiments). (P<0.05 and |log2 fold change|≥1.5). Genes associated with cortisol generation and cAMP signaling, such as *CYP17A1*, *MC2R*, *STAR*, *WNT9A*, *NR4A1*, *CREB5* and *LDLR* were upregulated in

ACTH-treated ACOs. (**B**) The top 20 pathways in GO and KEGG enrichment analysis with p.adj (minimal) of the differentially expressed genes of ACTH-treated ACOs versus CTRL ACOs (n=2 independent experiments). (**C**) The box graphs showed the hormone content secreted from ACOs. Supernatants were measured by mass spectrometry and revealed a significant increase of the analyzed steroid hormones in ACTH-treated ACOs compared to controls (nmol/L, n=3 independent experiments per group). *$p<0.05$; **$p<0.01$; ***$p<0.001$.

Supplementary Figure 4

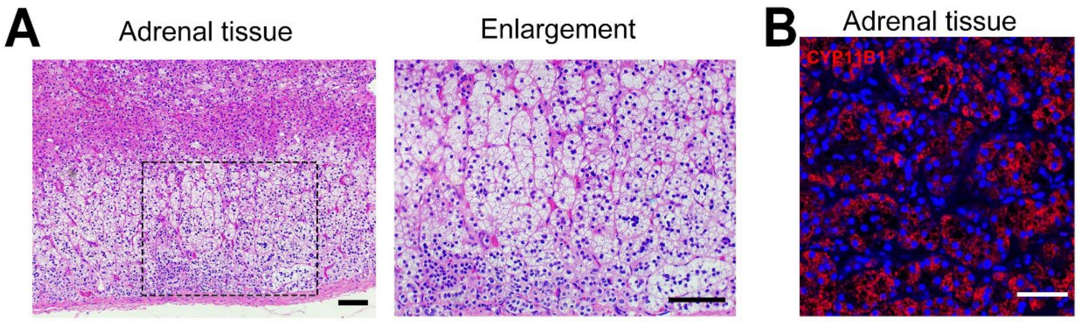

Supplementary Figure 4 | Histological analysis of human adrenal tissue. (**A**) H&E staining of human adrenal tissue. Black dashed curves outline the zF. Scale bar=100μm. (**B**) IF staining of CYP11B1 (red) and DAPI (blue) in human adrenal tissue. Scale bar=25μm.

Supplementary Figure 5

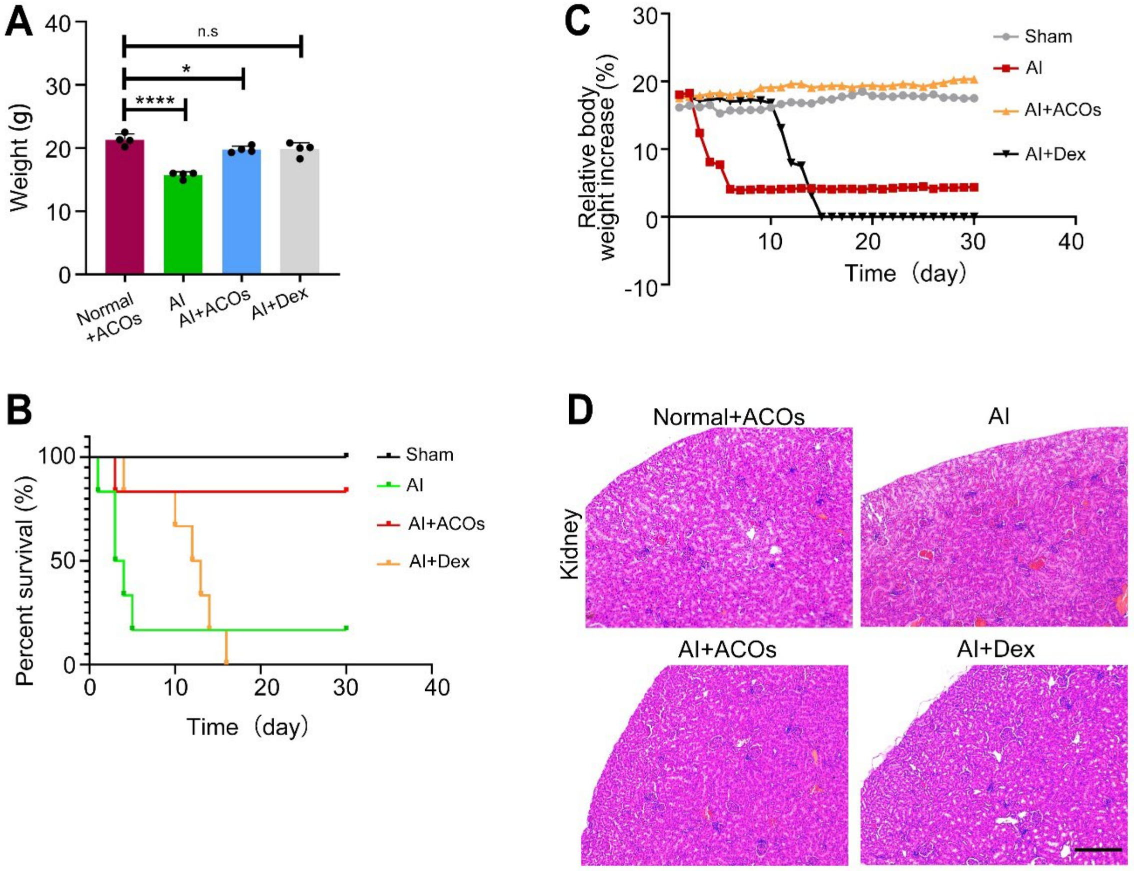

Supplementary Figure 5 | Human ACOs restore glucocorticoids homeostasis to rescue adrenalectomised mice. (A) Body weight of mice at six days after adrenalectomy or transplantation of P0 ACOs (n=4 independent experiments for each group), *$p<0.05$; ****$p<0.0001$. (**B**) Survival curve of Sham, AI, AI+ACOs and AI+Dex mice after transplantation of P2 ACOs (n=6 independent experiments for each group). (**C**) Body weight monitoring of Sham, AI, AI+ACOs and AI+Dex mice after transplantation of P2 ACOs (n=6 independent experiments for each group). (**D**) H&E staining of mouse kidney, at six days after adrenalectomy or transplantation of P0 ACOs (n=2 independent experiments for each group). Scale bar=100 μm.

Supplementary Figure 6

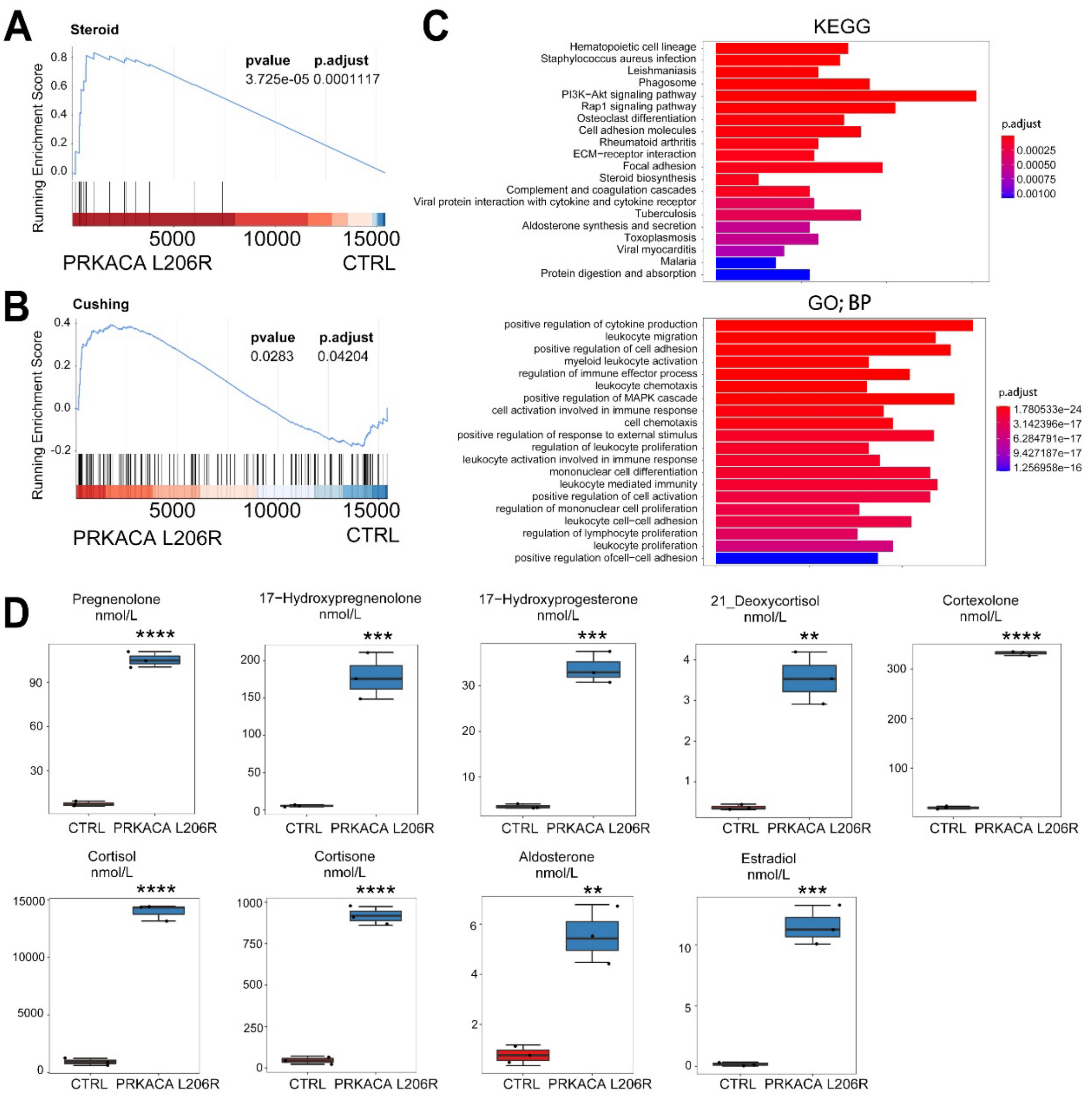

Supplementary Figure 6 | Introducing PRKACA L206R into human ACOs models the disease of cortisol-producing adenomas. (**A**) GSEA analysis showing enrichment of steroid synthesis pathway in PRKACA L206R ACOs versus control (CTRL) ACOs (n=2 donors per group). (**B**) GSEA analysis showing enrichment of Cushing's syndrome pathway in PRKACA L206R ACOs versus CTRL ACOs (n=2 donors per group). (**C**) Top 20 pathways in GO and KEGG enrichment analyses with p.adj (minimal) of the differentially expressed genes of PRKACA L206R ACOs versus CTRL ACOs (n=2 donors per group). (**D**) The box graphs showed the hormone content secreted from ACOs in supernatants, measured by mass spectrometry, were significantly increased in PRKACA L206R ACOs compared with their controls (n=3 donors per group). ***p*<0.01; ****p*<0.001; *****p*<0.0001.

Supplementary Table 1 | Patient Information.

No.	Age (year)	Gender	Figures for publication
1	34	M	Figure S1a-b
2	38	F	Figure S1a-b
3	38	M	Figure 1b-k, S1c-d
4	61	M	Figure 1b-k, S1c-d
5	58	M	Figure 1b-k, S1c-d
6	44	F	Figure 1b-k, S1c-d
7	37	F	Figure 1b-k, S1c-d
8	54	M	Figure 2a-d, 2f-g, S2a-d
9	48	M	Figure 2a-d, 2f-g, S2a-d
10	33	F	Figure 2a-d, 2e, S2a-d
11	48	M	Figure S2e-g
12	55	F	Figure S2e-g
13	57	F	Figure S2e-g
14	21	M	Figure 3, S3
15	43	F	Figure 3, S3
16	53	M	Figure 3, S3
17	42	F	Figure 4a-g
18	50	F	Figure 4a-g
19	28	M	Figure 4a-g
20	44	M	Figure 4h
21	35	M	Figure 4h
22	39	F	Figure 4h
23	31	F	Figure 4i-n
24	20	F	Figure 4i-n
25	57	M	Figure 4i-n
26	42	M	Figure 5, S5a and d
27	35	F	Figure 5, S5a and d
28	39	F	Figure 5, S5a and d
29	44	M	Figure 5, S5a and d
30	65	F	Figure 5, S5a and d
31	36	M	Figure 5, S5a and d
32	36	F	Figure 5, S5a and d
33	48	M	Figure 5, S5a and d

34	32	M	Figure S5b-c
35	42	M	Figure S5b-c
36	32	F	Figure S5b-c
37	43	F	Figure S5b-c
38	36	F	Figure S5b-c
39	38	M	Figure S5b-c
40	34	F	Figure 6, S6
41	47	M	Figure 6, S6
42	43	F	Figure 6, S6

Supplementary Table 2 | Antibodies used in this study.

Antibody Name	Company	Cat No.	Immunogen	Host species
SF-1	Santa Cruz	sc-393592	Human	Mouse, mAb
CYP11B2	ABclonal	A1443	Human	Rabbit
CYP11B1	Santa Cruz	sc-374096	Human	Mouse, mAb
CYP11B1	ABclonal	A15046	Human	Rabbit
CHGA	ABclonal	A9576	Human	Rabbit, mAb
SULT2A1	ABclonal	A8334	Human	Rabbit, mAb
SULT2A1	Santa Cruz	sc-166108	Human	Mouse, mAb
KI67	BD	550609	Human	Mouse, mAb

Note: mAb, monoclonal antibody.

Supplementary Table 3 | Primers for RT-qPCR.

Primer name	Sequence (5’→3’)
SHH-F	TGAACGGACCTTCAAGAGCC
SHH-R	GCAGGAGCATAGCAGGAGAG
DAX1-F	ATGGAGATCCCGGAGACCAA
DAX1-R	AAGAGCACGGTCCCTTTCAG
GLI1-F	TGGATCGGATGGGAGGTCTT
GLI1-R	ACCTCTGGCTCCTCCTGTAG
LGR5-F	CTCCCAGGTCTGGTGTGTTG
LGR5-R	GAGGTCTAGGTAGGAGGTGAAG
STAR-F	GGGAGTGGAACCCCAATGTC
STAR-R	CCAGCTCGTGAGTAATGAATGT
SF-1-F	CCGGCTACCACTACGGACT
SF-1-R	CTGCGTCTTGTCGATCTTGC
CHGA-F	TAAAGGGGATACCGAGGTGATG
CHGA-R	TCGGAGTGTCTCAAAACATTCC
CHGB-F	CGAGGGGAAGATAGCAGTGAA
CHGB-R	CAGCATGTGTTTCCGATCTGG
CYP11B2-F	TGACCGCAGGTTGCTTTCC
CYP11B2-R	AAGCCGAGACGACGACAGAC
AGTR1-F	GGCTATTGTTCACCCAATGAAGT
AGTR1-R	TGGGACTCATAATGGAAAGCAC
CYP11B1-F	AATACAGTGGTTGGAGAGGCATAG
CYP11B1-R	AAGCCGAGACGACGACAGAC
CYP17A1-F	GCTGCTTACCCTAGCTTATTTGT
CYP17A1-R	ACCGAATAGATGGGGCCATATTT
SULT2A1-F	CTGGGAAAGACGTTAGAACCC
SULT2A1-R	AAGTTGTGCTTTGTCCACTACAT
NR4A1-F	ATGCCCTGTATCCAAGCCC
NR4A1-R	GTGTAGCCGTCCATGAAGGT
CACNA1H-F	ATGCTGGTAATCATGCTCAACTG
CACNA1H-R	AAAAGGCGAAAATGAAGGCGT
LDLR-F	ACGGCGTCTCTTCCTATGACA
LDLR-R	CCCTTGGTATCCGCAACAGA
MC2R-F	CAAAGCCAAGGAGAGGAGCATTATT
MC2R-R	GGTGTTTGCCGTTGACTTACAGAAA
WNT11-F	GCCAATAAACTGATGCGTCTACA
WNT11-R	GTATCGGGTCTTGAGGTCAGC
ITPR3-F	CCAAGCAGACTAAGCAGGACA
ITPR3-R	ACACTGCCATACTTCACGACA

PLCB2-F	ATCCGGGATACTCGCTTTGG
PLCB2-R	CACCACCGTGAGTGTCTTCAG
PRKACA-F	CAGACTTCGGTTTCGCCAAGC
PRKACA-R	ACCAGTCCACGGCCTTGTTG
GAPDH-F	GAAGGTGAAGGTCGGAGTC
GAPDH-R	GAAGATGGTGATGGGATTTC

Supplementary Table 4 | Hormone and metabolite quantification in organoid supernatants.

ACTH induced hormone secretion (nmol/L)							
zG	CTRL1	CTRL2	CTRL3	ACTH1	ACTH2	ACTH3	*P* value
Corticosterone	3048.6	3666	4431.1	4036.7	4325.8	5136.2	0.204454
11-Dehydrocorticosterone	130.8	38.2	116.8	158.4	134.6	94.3	0.380566
Aldosterone	2.7	6.5	10.7	7.2	8.7	17.1	0.317947
zF							
Pregnenolone	17.106	14.3795	56.6677	88.4862	140.2351	269.0809	0.069349
17-Hydroxypregnenolone	1.6628	1.6348	2.4641	67.8753	49.8527	130.0091	0.029325
17-Hydroxyprogesterone	2.8175	2.5187	3.1781	47.9365	22.5269	41.9359	0.010702
21_Deoxycortisol	4.4784	11.4472	12.2496	64.5413	56.7751	74.2913	0.000583
Cortexolone	38.1905	64.9178	84.9751	546.2371	398.6242	367.9724	0.002707
Cortisol	681.4368	2178.409	2544.965	6699.0576	7250.1845	7520.1715	0.000982
Cortisone	19.4994	105.9092	79.4385	536.6009	1014.0744	700.8225	0.008699
zR							
Androstenedione	1.3199	0.4845	1.0091	165.9814	21.7559	86.9463	0.095459
Dehydroepiandrosterone	1.72387	1.72387	1.72387	49.2325	17.2387	44.7508	0.024134
Estrone	0.0398	0.0595	0.0131	28.0211	12.7733	17.2612	0.012958
Estradiol	0.0749	0.0788	0.0836	8.6321	5.1798	7.4737	0.002292
Medulla							
Metanephrine	BLD	BLD	BLD	BLD	BLD	BLD	ns
Normetanephrine	BLD	BLD	BLD	BLD	BLD	BLD	ns
Epinephrine	BLD	BLD	BLD	BLD	BLD	BLD	ns
Norepinephrine	BLD	BLD	BLD	BLD	BLD	BLD	ns

PRKACA L206R induced hormone secretion (nmol/L)							
zG	GFP1	GFP2	GFP3	PRKACA1	PRKACA2	PRKACA3	*P* value
Corticosterone	1089.5	1290.3	1390.5	1950.9	1828.3	2067.7	0.003513
11-Dehydrocorticosterone	16.9	16.1	19.8	75.1	57.1	73.6	0.000974
Aldosterone	0.3	1.1	0.7	4.4	6.7	5.4	0.002489
zF							
Pregnenolone	5.9066	9.3637	6.7495	100.4339	110.9274	104.8745	0.000006
17-Hydroxypregnenolone	4.3678	7.0813	5.4489	148.2811	210.8887	175.4945	0.000681
17-Hydroxyprogesterone	3.2045	4.1252	3.4255	30.7677	37.494	32.9207	0.000113

21_Deoxycortisol	0.3418	0.4494	0.3129	2.909	4.1932	3.5297	0.001044
Cortexolone	17.3268	23.2358	19.8395	333.9132	334.7388	327.0973	0.000001
Cortisol	628.8665	1244.526	931.3714	13153.851	14327.8168	14423.0567	0.000001
Cortisone	21.7056	71.2191	44.1249	973.661	860.889	917.5773	0.000001
zR							
Androstenedione	1.6778	1.873	1.7389	31.8315	43.7982	35.9795	0.00054
Dehydroepiandrosterone	1.0021	1.0826	0.892	15.2535	17.785	17.0277	0.000001
Estrone	0.1664	1.4156	0.7756	41.35	32.1188	34.7418	0.000218
Estradiol	0.0343	0.3616	0.1637	13.2366	10.053	11.2547	0.000264

Ang II induced hormone secretion (ng/mL)							
zG	CTRL1	CTRL2	CTRL3	AngII-1	AngII-2	AngII-3	*P* value
Corticosterone	1420.6861	1840.346	7291.2670	8913.0751	11032.2543	17236.5912	0.04716506
Aldosterone	1.5715368	2.692406	11.003697	36.166083	34.0213185	45.4728324	0.00190413
zF							
Pregnenolone	5.2287321	7.402580	11.780314	30.941502	35.8179337	61.0305363	0.02236931
Cortexolone	228.49535	307.1003	1237.7834	865.33169	778.527275	2743.23591	0.29184621
Cortisol	569.95635	673.3457	5475.1403	14315.962	14184.7082	12699.8684	0.00249079
Cortisone	38.465422	37.05683	350.16139	2128.4519	2436.9414	2080.76087	0.00017010
zR							
Androstenedione	0.929167	1.68912	4.887924	19.951464	16.8852991	16.0616132	0.00087005
Dehydroepiandrosterone	0	0	0	1.9432325	0	1.57104264	0.12055884
Estrone	0.441110	0.84919	0.349229	497.44475	494.909363	105.332248	0.04860034
Estradiol	0	0	0	72.85704	74.730109	32.658118	0.01189025

Note: BLD, below limit of detection.

Supplementary methods

Organoids passage. Organoid pellets were dissociated in 2 ml TrypLE Express (Invitrogen) via 1–5 min incubation at 37°C with pipette shearing. After adding 2 ml PBS and centrifuging at 300 g, fragments were resuspended in ACO-medium, seeded in 24-well low-adhesion plates to reform organoids, and passaged every 7 days. P2–P3 organoids were used for bulk and single-cell RNA-seq to avoid passaging effects.

Transmission electron microscopy. Organoids were collected and washed twice with PBS, fixed with 2.5% glutaraldehyde for more than 2 h, and then prepared for transmission electron microscopy.

ACTH and angiotensin II stimulation *in vitro*. To assess whether organoids can respond to ACTH and angiotensin II stimulation, organoids were incubated with medium containing $ACTH_{1–39}$ (3nM, Beyotime) or angiotensin II (3nM, MCE) for 48 h. Organoids were collected for RNA-sequencing. Cell culture supernatants were collected and used for hormone content determination.

Lentiviral vector construction and production. For lentivirus vector construction, the sequence of human PRKACA L206R was cloned into the pLVX-P2A-EGFP vector. For lentivirus production, the triple-plasmid transfection was performed. HEK293T cells were transfected with three plasmids (Core plasmid: psPAX2: pMD2.G=7:5:2) using VigoFect (Vigorous) according to the manufacturer’s instructions.

RNA Sequencing Analysis. Total RNA from control, ACTH-stimulated, or PRKACA L206R organoids was isolated using the RNAprep Pure Micro Kit (Tiangen Biotech) and then sent to the company (Xuran Biotechnology) for quality control and sequencing. RNA-seq libraries were prepared on the Illumina HiSeq platform and more than 40 million reads were obtained for each sample. The raw sequencing data quality was checked using FASTQC (https://www.bioinformatics.babraham.ac.uk/projects/fastqc/). After trimming, the raw sequences were mapped and assembled to human genome (GRCh38) through HISAT2 v2.2.1/StringTie v2.2.1 with default parameters. Downstream analyses were

processed in R v4.1.3. In brief, DESeq2 v1.34.0 was used to identify DEGs. Genes with a log2 fold change greater or lesser than 1.5, and an adjusted *p*-value less than 0.01, were considered as differentially expressed genes (DEGs). ClusterProfiler v4.10.0 was utilised for conducting GO and KEGG analyses, as well as GSEA employing hallmark gene sets obtained from the GSEA database. Gene expression heatmaps were generated using pheatmap v1.0.12.

Hormone quantification. Supernatants from treated organoids and mouse serum were collected for steroid hormone quantification by mass spectrometry (Metabo-Profile). Briefly, 100 µL of culture supernatant was transferred to a centrifuge tube, mixed with 50 µL acetonitrile containing deuterated internal standards and 1 mL MTBE, vortexed for 30 s, and centrifuged at 18,000 g for 10 min. The supernatant was dried under nitrogen at 60 °C, redissolved in 70 µL 20% methanol, and analyzed by UPLC-MS/MS (ACQUITY UPLC-Xevo TQ-S, Waters).

Control and PRKACA L206R organoid culture. For human adrenocortical organoids, 20 µL PRKACA L206R lentivirus (pLVX-P2A-EGFP lentivirus was used as control) were co-incubated with fragmented organoids in one well of a 24-well plate in 200 µL ACO-medium for six hours at 37°C, subsequently seeded in a 24-well low adhesion plate. Three days later, the organoids were treated with 1 µg/ml puromycin until they became resistant to puromycin. Expression of *PRKACA L206R* in control and PRKACA L206R organoids were validated using qRT-PCR. Quantification of organoids area: ACOs were generated from three different biological donors for both the control and PRKACA-L206R groups. For each sample, three bright-field images were captured at random locations after 15 days of culture, resulting in a total of 9 images per group. Every single, fully visible organoid across all 9 images for each group was identified and its area was quantified using ImageJ. The data from all organoids measured for each condition were then pooled for statistical analysis and visualization using GraphPad Prism.
